# Social microbiota and social gland gene expression of worker honey bees by age and climate

**DOI:** 10.1038/s41598-022-14442-0

**Published:** 2022-06-23

**Authors:** Kirk E. Anderson, Patrick Maes

**Affiliations:** 1 Carl Hayden Bee Research Center, USDA Agricultural Research Service Tucson, 2000 E. Allen Rd., Tucson, AZ 85719 USA; 2 Department of Entomology and Center for Insect Science, University of Arizona, Tucson, AZ USA

**Keywords:** Ecology, Evolution, Microbiology, Molecular biology

## Abstract

Winter forage dearth is a major contributor to honey bee colony loss and can influence disease susceptibility. Honey bees possess a secretory head gland that interfaces with the social environment on many levels. During winter or forage dearth, colonies produce a long-lived (diutinus) worker phenotype that survives until environmental conditions improve. We used a known-age worker cohort to investigate microbiome integrity and social gene expression of workers in early and late winter. We provide additional context by contrasting host-microbial interactions from warm outdoor and cold indoor environments. Our results provide novel evidence that social immune gene expression is associated with worker longevity, and highlight the midgut as a target of opportunistic disease during winter. Host microbial interactions suggest opportunistic disease progression and resistance in long-lived workers, but susceptibility to opportunistic disease in younger workers that emerged during the winter, including increases in Enterobacteriaceae, fungal load and non-core bacterial abundance. The results are consistent with increased social immunity, including host associations with the social microbiota, and a social immune response by long-lived workers to combat microbial opportunism. The cost/benefit ratio associated with limited expression of the diutinus phenotype may be a strong determinant of colony survival during winter forage dearth.

## Introduction

Social insects are under strong selection to evolve antibiotic mechanisms that result in group hygiene, or social immunity^1,2^. There are fascinating social insect symbioses evolved to control the abundance of particular microbial species throughout the nest, hive or social environment^3–6^. The social context of disease susceptibility involves multiple factors that shape the evolution of life history, including resident microbial symbionts and resistance to opportunistic disease^7–10^. Many opportunistic disease states are associated with changes in core hindgut structure or the overgrowth of native colony microbiota, highlighting the importance of microbiome integrity or taxonomic membership in disease susceptibility. How, when and where opportunistic pathogens can invade the host depends in part on their ability to occupy sub-optimal or fringe niches with consistent host exposure, to form relationships with, or outcompete other resident microbes, and to evade host defenses^8,11,12^. A primary function of gut microbiota in eukaryotes is protection from pathogens, and many factors may weaken the core microbiota rendering the host organism susceptible to disease^12–16^. In humans, the skin and nasal pharyngeal microbiome are considered the first line of defense against pathogen colonization and infection^17^. Analogous to these protective human niches, the honey bee is host to a variety of microbiotas associated with a type of “social skin” that occurs throughout the colony and hive^4,13,14,18,19^. This social microbiota is associated with social nutrient processing and information sharing including the structurally complex worker mouthparts that perform these tasks and a secretory hypopharyngeal gland (HPG) in the worker head. The HPG produces a highly nutritious and bioactive jelly substance containing various cocktails of pro-oxidants, antioxidants and antimicrobial peptides that interface constantly with the microbiota and social network^4,14,18^.

A recently emerged model for gut microbiome studies, the honey bee worker hindgut microbiota is highly predictable by both taxonomy and structure, comprised primarily of five core phylotypes represented in every study^7,12,20–24^. In the rectum, *Lactobacillus* firm5, *Lactobacillus* Firm4, *Bifidobacterium asteroides* dominate, while *Snodgrassella alvi*, *Gilliamella apicola* and *Lactobacillus* Firm5 dominate the ileum. The structure and function of the hindgut microbiota is essential to the health of the individual worker and colony. The core phylotypes occur throughout the colony and hive environment, facilitating generational transmission^25–27^. Similar to the hindgut, a variety of niches throughout the colony and hive also contain a unique and predictable set of phylotypes^4,19,22,28–30^. The honey bee maintains a strict relationship with the microbes in its hindgut, and we predict similar functional pressure exists to control the microbiota throughout the interactive social environment.

Honey bee life-history and aging is extremely plastic; queens can live for years, and workers also exhibit considerable longevity differences associated with age, task, winter survival and forage dearth^31–34^. Similar to other model systems, the hindgut microbiota tracks the physiology of honey bee behavioral and reproductive phenotypes associated with age and nutritional state^30,35^. Worker bees acquire their highly structured hindgut microbiota in the first 1–4 days of adult life concurrent with the consumption of beebread; pollen, honey and worker secretions^25,26^. Host digested beebread is converted to an internal storage molecule, *Vitellogenin* (*Vg*), a large lipo-glyco-phosphoprotein. The role of *Vg* varies by tissue and caste and is associated with antioxidant, antimicrobial, and anti-inflammatory properties^36^. Workers raised in stressed conditions do not establish a typical gut microbiota, and show deficient central metabolism including decreased insulin signaling and *Vg* production^27^, and as a social consequence, deficient HPG secretions.

The HPG can express a broad variety of nutritional and antimicrobial cocktails in response to social needs. Jelly contains antimicrobial peptides and social hygienic enzymes *glucose oxidase* (*GOX*), and *superoxide dismutase* (*SOD*). Mixed with glucose in honey, *GOX* produces gluconic acid and hydrogen peroxide. The honey bee social environment is coated with *GOX* and H_2_O_2_ providing a generalized social immune barrier^1,37–39^. Phenotypic plasticity within the worker caste is reflected in HPG gene expression; young nurses produce copious amounts of nutrient rich jelly, but the HPG shrinks in older foragers and secretes enzymes associated with honey processing^40,41^. The HPG of workers can resume past physiological states associated with youth, or shift forward to assume the task of an older bee^42^. The gland can also react more proximally to colony-level challenge associated with a variety of social pressures like emergency queen rearing^43,44^. In addition to providing shared nutrition, jelly produced in the HPG may transmit immune training molecular patterns across generations, extend life expectancy, modulate microbiota structure and prevent opportunistic disease^44–46^. Gene expression associated with individual and social immunity is costly, and only generated by well-nourished individuals^38,47^. Because internal *Vitellogenin* stores over winter rely on nutrition in the fall, the availability of pollen on the landscape is a major factor in brood production, colony growth and disease resistance^48^.

Honey bee colonies respond rapidly to environmental conditions. The proximal cue of artificial rainfall causes a shift to conservation physiology in a matter of hours^49^. Similarly, forage dearth and persistent low temperatures shift physiology towards colony thermoregulation and resource conservation including production of the diutinus worker phenotype, defined by worker longevity and conservation physiology. Diutinus workers consume and digest pollen in the fall, or at the beginning of a pollen dearth, then conserve the nutrition internally to provide for the growing colony when environmental conditions improve. With the coming of winter, pollen diversity and nutrition disappear completely across the northern landscape, and decrease drastically on the southern landscape. While the diutinus phenotype is well documented in northern climates, there is little to no data on its expression during mild winter conditions typical of the southern US including Texas, Florida and California where an economically significant number of commercial bee colonies overwinter annually. These mild winters involve a variety of indistinct or conflicting environmental cues, including warm daily temperatures, and the availability of pollen and nectar on the landscape. Subsequently, brood rearing over winter commonly discontinues in predictably cold (Northern) climates, but continues at significantly reduced levels in southern climates, stimulating foraging behavior throughout the winter^50,51^. The presence of brood in the hive environment accelerates both behavioral and cellular senescence among worker bees^31^. After approximately 10 days of foraging, workers experience a sharp increase in mortality^31^.

In continuously cold winter climates, the cohort of workers emerging in early winter express the diutinus phenotype, and can live 8X longer (240 days) than a worker during spring/summer colony growth^52,53^. Although a critical point in the life cycle, variation in gut and colony microbiota and physiology that accompanies this process is relatively unknown, as is the social immune status of diutinus workers during winter^54,55^. A recent investigation of the diutinus hindgut microbiota implied healthy physiology in cold, climate-controlled (7 °C) indoor conditions, in contrast, greatly reduced longevity and the proliferation of opportunistic bacteria were associated with the southern winter^8^. However, the putative opportunistic microbes were at extremely low abundance in the hindgut relative to the core microbiota. In this contribution, we investigate these same samples more deeply by examining the change in worker mouth and midgut microbiotas, and detailing the associated change in HPG gene expression. Like other examples from social insects^5,6^, we hypothesize that HPG social secretions nurture symbionts native to the alimentary tract, but are negatively associated with opportunists in the social and gut environment.

## Methods

**Figure 1:**
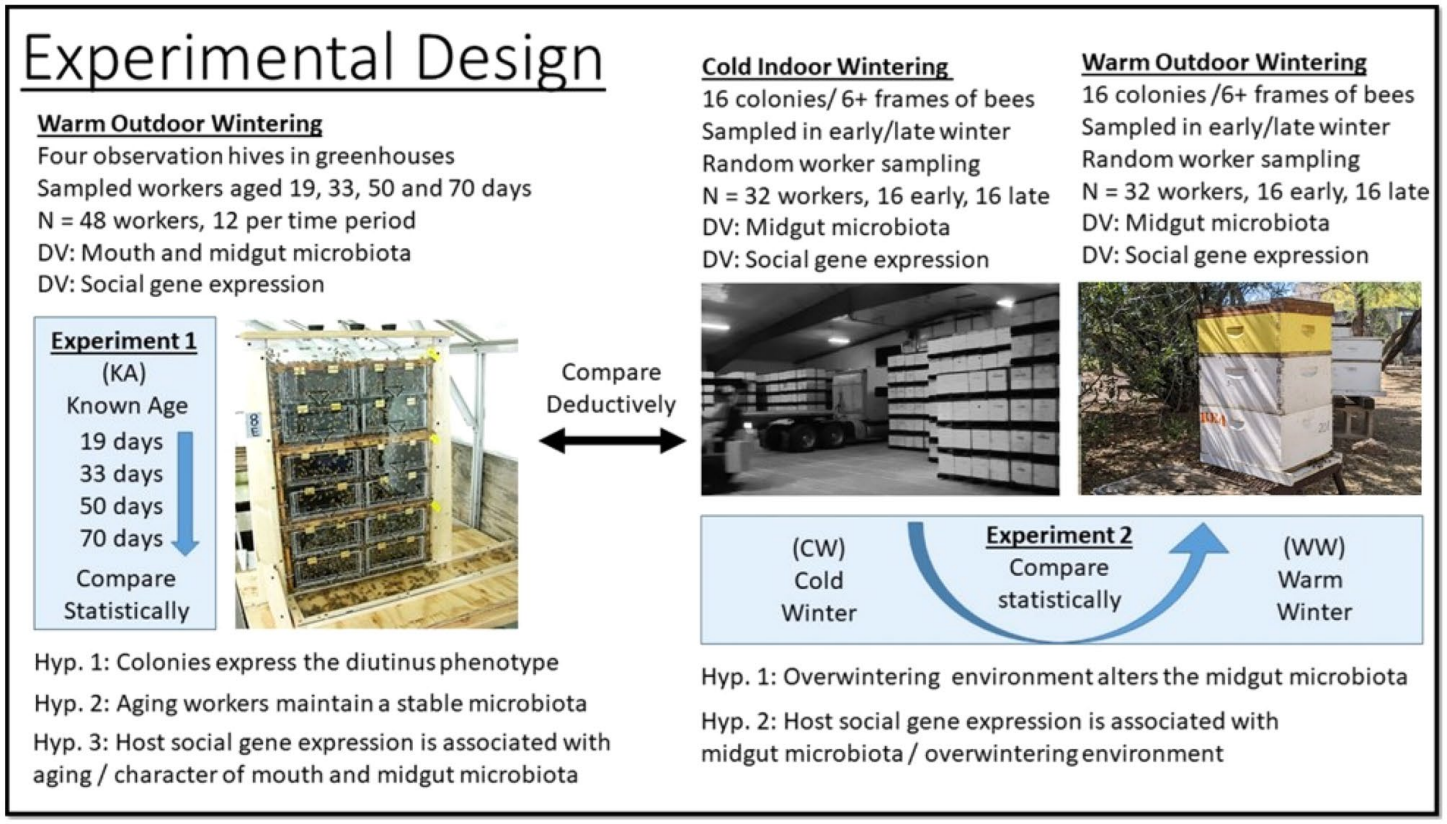
Experimental design, sampling details and hypotheses. The blue boxes describe the manipulations performed in this study. DV is Dependent Variable. Cold winter warehouse photo provided by Agri-Stor Companies of Idaho.

To gain an understanding of host-microbial interaction during winter, we performed two separate but related experiments (Fig. 1). We designed the first experiment to provide perspective on the relationships of mouth and midgut microbiota with HPG gene expression. We quantified HPG gene expression and the microbiotas associated with worker longevity from colonies kept in warm winter conditions. The second experiment compared HPG expression and various microbiota characteristics of the midgut associated with full-sized colonies placed in cold indoor climate-controlled wintering, and warm outdoor wintering. These results are associated with a published companion paper that sequenced the ileums and rectums from the same experimental samples^8^. Briefly, those results revealed statistically indistinguishable hindgut microbiotas in early winter, and little change in the hindgut microbiota of worker bees during winter regardless of climate or age. However, the authors suggested the potential for compromised host physiology in warm southern climates, revealed by low worker longevity from early to late winter, and significantly increased fungal load correlated with an increase in putatively opportunistic Enterobacteriaceae. The present manuscript more fully explores the microbiomes and host response associated with microbial opportunism and fungal growth in aging worker bees during winter dearth.

### Experiment 1: known age cohort

We used four observation hives to follow a known age (KA) cohort and validate chronological age. To control for age in the observation hive, newly emerged winged adults were sourced from brood frames of 20 healthy colonies. Late stage pupae emerged naturally from their natal frames over a period of ≤ 24 h while housed in a humidity (50%) and temperature-controlled (35 °C) room. All newly emerged bees were collected into a single container and randomized prior to colony assignment. We painted the thoraces of 2,000 newly emerged bees and divided them equally among four observation colonies. Observation hive colonies comprised of three or four frames containing brood, plentiful honey and beebread, were kept in greenhouses at the Carl Hayden Bee Research Center, Tucson AZ with exposure to diurnal cycles, access to the foraging environment and a small space heater for rare nights below freezing. Adult worker bees were introduced into observation hives on December 3rd, then sampled from Dec 22nd 2015–Feb 10th 2016. Samples of marked bees were collected at 19, 33, 50 and 70 days of age, and we analyzed 12 workers per age group (*n* = 48). Our 19 Day-old sample represents the age when workers have expelled the pollen from their guts and transitioned to foraging, while 33 days is the mean expected longevity of foragers in spring/summer. In the companion study, we estimated that only 10% of introduced bees survived until 50 days of age^8^. Thus, our 50 and 70-day-old samples were comprised of the longest-lived bees relative to average life expectancy in southern climates. From this sample set, we quantified the change in HPG gene expression in early and late winter of host genes related to reactive oxygen species, and antimicrobial peptide (AMP) expression. The ileum and rectum microbiota from these same worker samples (KA) was remarkably stable from 19 to 70 days of age while fungal load and bacterial diversity decreased significantly during winter in the hindgut^8^.

### Experiment 2: overwintering climate comparison

In a second experiment, we hypothesized that the midgut microbiomes associated with warm vs. cold winter conditions would differ due to task differences experienced by the colony under different climatic conditions. We compared the midgut microbiota of colonies from southern outdoor climates to colonies kept indoors throughout the winter in cold climate controlled conditions. All colonies in this experiment had access to honey and beebread during winter, but the cold winter colonies became broodless, while the warm winter colonies continued to forage and rear small numbers of brood throughout the winter^50,51^. All full size colonies in both environments were 6 + frames of bees at the beginning of the experiment with no signs of disease.

The warm overwintering (WW) environment was the Santa Rita Experimental Range in southern Arizona 31°46′38″N, 110°51′47″W. We collected worker samples in mid-December 2015 and mid-February 2016. We placed cold winter (CW) colonies in a climate controlled storage facility at a constant 7 °C and 25% relative humidity with no access to environmental cues. Worker bees were sampled just prior to entering the cold storage facility in Firth, Idaho, USA, 43°18′45″N, 112°09′20″W in early winter, mid-October 2015, and directly after they were removed from cold storage and transported to the almond orchards in late winter, mid-February 2016, near Snelling, California, USA, 37°31′33″N, 120°31′52″W. From the edge of the brood nest, we sampled worker bees from 16 colonies per time point, per climate, performing molecular analysis on 64 total colony samples. The hindgut microbiotas of early winter bees did not differ by climate/locale but fungi increased significantly during winter in the hindguts of WW samples, concurrent with a slight but significant increase in an unknown *Gilliamella* spp. and Enterobacteriaceae^8^.

### DNA analysis

We performed microbial DNA analysis on the mouthparts and midguts quantifying total bacteria and fungi. Immediately after being removed from − 80 °C individual bees were surface sterilized and dissected in 95% ethanol using sterile forceps and dissection scissors. We immediately placed all tissues into a 2 ml bead beating tube containing ~ 100 μl of 0.1 mm silica beads and 300 μl of 1X TE buffer (10 mM Tris–HCl, 1 mM EDTA) and immediately frozen on dry ice. All dissected tissues were stored at  −80 °C. Prior to DNA extraction, each sample was bead beaten for a total of 2 min in 30 s intervals. To each sample, 100 μl lysis buffer (20 mM Tris–HCl, 2 mM EDTA, 5% Triton X-100, 80 mg/ml lysozyme, pH 8.0) was added and the samples were incubated at 37 °C for 30 min. Total genomic DNA was extracted using a Fermentas GeneJet Genomic DNA Purification Kit (#K0722) following the protocol for gram-positive bacteria. Miseq amplification and analysis was performed as in^8^. We estimated the size of the bacterial/fungal communities in the ileum and rectum using degenerate primers^56,57^. We amplified the 16 s gene template using forward primer 27F (5’-AGAGTTTGATCCCTCAG-3’) and reverse primer 1522R (5’-AAGGAGGTGATCCAGCCGCA-3’). The 18 s gene template was amplified using forward primer PanFungal_18S_F (5’-GGRAAACTCACCAGGTCCAG-3’) and reverse primer PanFungal_18S_R (5’-GSWCTATCCCCAKCACGA-3’). We created plasmid vectors using Invitrogen’s pCR^TM^2.1 TOPO™ cloning vectors per the manufacture’s specifications.

### Microbiota sequencing

For all samples (Experiment 1; *n* = 48, Experiment 2; *n* = 64), the V6–V8 region of the 16S rRNA gene was amplified using universal (degenerate) PCR primers 799F (acCMGGATTAGATACCCKG) and bac1193R (CRTCCMCACCTTCCTC). We amplified DNA using the HotStarTaq Plus Master Mix Kit (Qiagen, Germantown, MD, USA) under the following conditions: 94C for 3 min, followed by 28 cycles of 94 °C for 30 s, 53 °C for 40 s and 72 °C for 1 min, with a final elongation step at 72 °C for 5 min. We used the PCR products to prepare DNA libraries following Illumina MiSeq DNA library preparation protocol. Sequencing was performed at the University of Arizona Genetics Core (UAGC) on a MiSeq following the manufacturer’s guidelines. Resulting sequences were processed using MOTHUR v1.43^58^, and command lines are listed in Supplementary Table S1.

### Expression analysis

RNA was extracted from the hypopharyngeal glands of all 112 workers from both experiments. Heads were surface sterilized and dissected in RNAlater™ using sterile forceps and dissection scissors. All samples were immediately placed into a 1.5 ml centrifuge tube containing 200 µL of Fermentas Lysis Buffer supplemented with β-mercaptoethanol and immediately frozen on dry ice. All samples were stored at −80 °C. Total RNA was extracted using a Fermentas GeneJet RNA Purification Kit (#K0732) following the protocol for Total Insect RNA Purification. Complementary DNA (cDNA) was generated using the Thermo Scientific RevertAid First Strand cDNA Synthesis Kit (#K1622) following the First Strand cDNA Synthesis protocol. We quantified the expression of eight genes relative to the housekeeping gene β-actin: three antimicrobial peptides; *Hymenoptaecin, Defensin-1* and *Abaecin*; three antioxidant genes; *Catalase, Zn/Cu Superoxide Dismutase* (*Zn/CuSOD*) and *Mn Superoxide Dismutase* (*MnSOD*); the redox “social immunity” enzyme *Glucose Oxidase* (*GOX*) and finally, *Vitellogenin* (*Vg*) as a marker of nutritional state. Briefly, all reactions were performed in a 12 μl volume (6ul of Bio Rad iTaq Universal SYBR Green Supermix, 0.5 μl of 10 μM forward and reverse primer [see^59^ for primer details], 3 μl of molecular grade water and 2 μl of template). Cycle conditions were 95 °C for 30 s and then 40 × of 95 °C for 20 s and 60 °C for 30 s^59^. Amplification was normalized to β-actin expression.

### *Nosema* quantification

The midgut is the site of *Nosema* infection, a highly specialized fungal microsporidian that is a ubiquitous opportunist of honey bee midguts. To determine *Nosema* infection status in the midgut we ran a modified version of the qPCR protocol reported in^60^. For each 10 µl reaction we used 5 µl of Luna qPCR master mix (New England Biolabs), 0.25 µM of each primer, 1 µl of template and 3.5 µl molecular grade water. We used *N. cerana*e specific primers NcF (AAGAGTGAGACCTATCAGCTAGTTG) and NcR (CCGTCTCTCAGGCTCCTTCTC) and *N. apis* specific primers NaF (GCCCTCCATAATAAGAGTGTCCAC) and NaR (ATCTCTCATCCCAAGA). To confirm efficiencies reported in^60^ we ran a temperature and concentration gradient qPCR with a known *Nosema* rich DNA sample. Amplification efficiency was calculated by the CFX manager software and were close to 100% (97.1% and 98.3% respectively). Using a CFX96 real time system (BioRad, Hercules, CA) we ran the following thermocycler program: 95 °C for 3 min followed by 40 cycles of 95 °C for 10 s and 63 °C for 30 s. Each sample and negative controls were run in triplicate. Using the same reagent mixture (with a 57 °C annealing/extension step), we used β-actin specific primers ActinF (TGCCAACACTGTCCTTTCTG) and ActinR (AGAATTGACCCACCAATCCA). We used the average of the three reactions to determine *N. ceranae* load relative to β-actin expression. We compared the relative qPCR measures to a previous microscopic assessment of *Nosema* abundance based spore counts from midgut samples of (CW) workers maintained indoors at 7 °C and 25% RH.

### Statistical analysis

We analyzed microbial community structure and abundance with two different approaches: MANOVA performed on centered log ratios and non-parametric Wilcoxon tests. The MANOVA accounts for microbiota structure while the Wilcoxon test examines OTU abundance normalized by bactquant results without regard to microbiota structure. We compared differences in the microbial community structure by known chronological age (KA samples) as well as within, and between WW and CW environments. To allow the use of parametric multivariate analyses^61^, we converted bacterial abundances to ratio abundances among all OTUs^62^ using the software CoDaPack’s centered log-ratio (CLR) transformation^63^. We compared microbiota structure by chronological age (known age cohort) and season (pre/post winter) within and between climates using one-way MANOVA. Pillai’s Trace test statistic was used for all MANOVA’s to account for deviations in normality and homogeneity of covariance. Subsequent univariate tests followed by FDR correction for multiple comparisons were used to explore differences between dependent variables (OTU’s).

We compared total microbial load for bacteria, fungi and *Nosema* using one-way ANOVA (FDR corrected for multiple comparisons). All analyses were conducted in either JMP_v13 (JMP_1989–2007) and/or SAS_v9.4 (2013). Pillai’s Trace test statistic was used for all MANOVAs to account for deviations in normality and homogeneity of covariance. Statistically significant MANOVA results were further analyzed with pairwise ANOVA tests followed by FDR correction for multiple comparisons. We analyzed total cell number using pairwise Wilcoxon tests (Steel–Dwass correction for multiple comparisons). To normalize to absolute abundance, the proportional abundance of OTUs returned by amplicon sequencing was multiplied by the total bacterial 16S rRNA gene copies determined with qPCR for each individual tissue type. To account for variability in 16S copy numbers across taxa the 16S total was divided by the number of 16S rRNA gene copy numbers present within each bacterial genome. The remaining OTUs (14–6294) were summed, and assigned a gene copy number of 4.2, the average for all bacteria^64^. This value represents a general measure of non-core bacterial abundance as it contains > 99% of the OTUs. To examine hypotheses of host microbial interaction, we generated Spearman's rank correlation coefficient among the dependent variables independently for each treatment condition, and across the entire data set.

## Results

### Experiment 1: known age cohort microbiota

**Figure 2:**
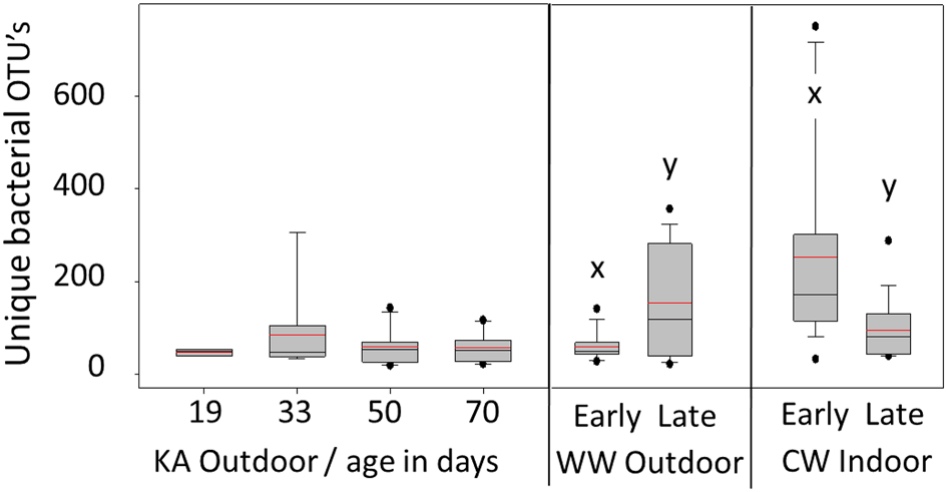
Bacterial diversity in midguts of worker bees depicted as the number of amplicon sequence variants or unique OTUs. Known age (KA) bees did not differ in diversity by age class from early to late winter. Diversity increased significantly in the warm winter (WW) outdoor environment (t_30_ = 2.89, *p* = 0.007) and decreased significantly in the cold winter (CW) indoor environment (t_32_ = 2.89, *p* = 0.008). Grey boxes contain 50% of the variation, whiskers contain 90%, and the dots represent the range. The horizontal red bar is the mean and black is the median. Boxplots created with SigmaPlot (Systat Software, San Jose, CA).

**Figure 3:**
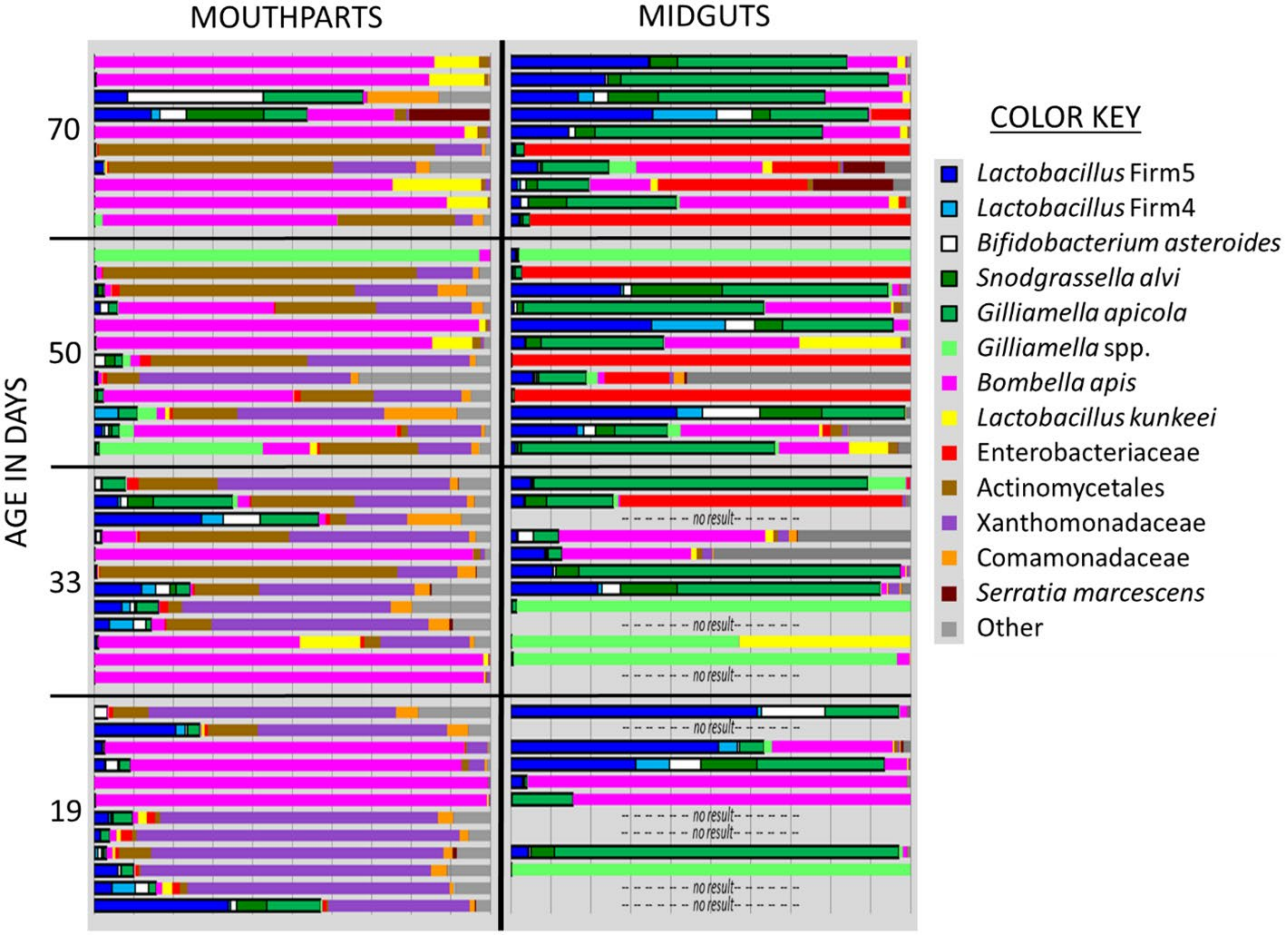
Relative OTU abundance for mouthpart (*n* = 46) and midgut (*n* = 38) microbiotas of KA worker bees maintained in Tucson AZ, USA. The five core hindgut bacteria at the top of the key are outlined with a black border. Ages on the y-axis coincide with sampling dates; Newly emerged bees were marked and introduced to colonies in Early December, then sampled in late December at 19 days old, mid-January at 33 days old, late January at 50 Days old and mid-February at 70 days old. Significantly larger microbiotas are associated with *B. apis* (Pink) on the mouthparts, and Enterobacteriacae (Red) and *Gilliamella* spp. (GFP green) in the midgut.

To compliment previous results from the hindgut^8^, we deep-sequenced the mouthpart and midgut microbiota of aging worker bees over wintered in southern Arizona. As the winter progressed, we sequenced known age (KA) workers at 19, 33, 50 and 70 days of age resulting in a total of 46 mouthpart and 38 midgut microbiota libraries (Fig. 1, Experiment 1). Bacterial diversity on the mouthparts was greatest in 33-day-old bees and decreased in older bees (Fig. 2). The mean number of unique OTUs detected on the mouth did not differ from that detected in the midgut and did not change with age (mouth = 57, midgut = 62). The mouth and midgut environments contained many of the same bacteria, including all five of the core hindgut phylotypes, but showed very distinct microbiotas (Fig. 3). Based on a Wilcoxon rank sum test comparing 19/33 day old bees to 50/70 day old bees, bacterial load remained steady on the mouth with age, but increased in the midgut (W_46_ = 166, *p* = 0.03). In contrast, total fungal load decreased slowly and significantly on the mouthparts of aging bees (W_46_ = 114, *p* = 0.0004) but remained steady in the midgut.

The mouthpart microbiota was comprised of *Bombella apis* (previously referred to as *Parasacharribacter apium* or Alpha 2.2*),* Actinomycetales, Xanthomonadaceae, *Delftia* (Comamonadaceae), an unknown *Gilliamella *spp.*,* and *Lactobacillus kunkeei.* Enterobacteriaceae occurred at low abundance on 95% of the mouthparts, and Actinomycetales, Xanthomonadaceae and *Delftia* had 100% prevalence and relatively even abundance. *B. apis* and *L. kunkeei* were both prevalent on the mouth but varied considerably in relative and normalized abundance (Fig. 3). The mouth microbiota of the aging bee (KA) differed from early to late winter. On the mouthparts of aging bees, Actinomycetales and the unknown *Gilliamella* spp. increased while Xanthomonadaceae decreased from twenty-six to nine percent of the total mouthpart community. Relative abundance of Enterobacteriaceae on the mouth also decreased significantly with age (Supplementary Table S1). We examined the aging bees by predicted task, assuming 19–33 days old bees were potential foragers or young workers and 50–70 day old bees were long-lived workers. According to this grouping, non-core bacterial abundance on the mouth decreased with age, while the relative abundance of *L. firm5, L. firm4* and *B. asteroides* on the mouth also decreased with age (Fig. 3).

The aging worker midgut supported Enterobacteriaceae, *Bombella apis,* an unknown *Gilliamella* spp., *L. kunkeei* and the five core hindgut bacteria. The triad signature of Actinomycetales, Xanthomonadaceae, and *Delftia* was present in nearly every midgut at low relative abundance (Fig. 3). All five of the core hindgut bacteria increased with age (Fig. 3); *Snodgrassella alvi* and *G. apicola* increased significantly, while *L. firm5* and *B. asteroides* also increased, trending towards significance (Supplementary Table S1). A putative midgut opportunist; Enterobacteriaceae also increased markedly with age. Although at low absolute abundance in KA midguts, the relative abundance of both *Delftia* (Comamonadaceae) and Xanthomonadaceae decreased with age during winter. Both Lachnospiraceae and *Serratia* were at low abundance in the KA mouthparts and midguts, present in < 50% of samples (Supplementary Table S1).

*Nosema ceranae,* a ubiquitous midgut pathogen, was at generally low absolute abundance in the midgut of aging bees. Many samples from all KA age groups did not rise above the limit of detection. Nonetheless, *N. ceranae* levels differed significantly among age classes, peaking at 33 days of age and declining thereafter. Young (19-day-old) and the oldest (70-day-old) bees contained significantly less *N. ceranae* than 33-day-old bees (Supplementary Table S2).

### Experiment 1: known age cohort gene expression

To test the hypothesis that social gene expression of long-lived worker honey bees affects the colony and/or gut microbiome, we examined associations of HPG gene expression with the aging microbiota (KA samples) of the mouth and midgut in early and late winter (Table 1, see Supplementary Table S3 for details). The relative HPG expression of five genes regulating oxidative state and AMP production increased significantly with age (Table 1). ROS associated gene expression had a strong and significant negative association with both non-core bacterial abundance and fungal load on the mouth. Actinomycetales increased significantly with age on the mouth and was positively associated with ROS gene expression. Xanthomonadaceae decreased significantly with age on the mouthparts, and was negatively correlated with ROS associated gene expression (see Supplementary Table S4 for details).

**Table 1:** Change (Δ) in hypopharygeal gland gene expression over winter.

Change (Δ) during winter	Warm winter known age*			Warm winter random sample			Cold winter random sample		
Gene	Z	*p*	**Δ**	Z	*p*	**Δ**	Z	*p*	**Δ**
**Nutritional state**									
Vitellogenin	0.69	0.49	**NC**	− 2.00	*0.05*	**↓**	0.42	0.68	**NC**
**Antimicrobial peptides**									
Hymenoptaecin	2.69	*0.007*	**↑**	− 1.52	0.13	**↓**	1.34	0.18	**↓**
Defensin-1	− 0.67	0.53	**NC**	1.62	0.11	**↓**	2.18	*0.03*	**↓**
Abaecin	2.87	*0.004*	**↑**	− 2.21	*0.03*	**↑**	1.42	0.16	**↓**
**Oxidative state**									
Glucose Oxidase	2.80	*0.005*	**↑**	0.02	0.98	**NC**	2.05	*0.04*	**↓**
Cu/ZnSOD	5.14	*0.0001*	**↑**	− 0.58	0.56	**NC**	− 0.18	0.86	**NC**
MnSOD	5.57	*0.0001*	**↑**	1.10	0.27	**NC**	− 0.66	0.51	**NC**

*The change (Δ) in expression during winter compares 19/33 days to 50/70 days of age. In the warm winter and cold winter environments, we compared randomly sampled in-hive workers in early vs. late winter. See Table S3 for details.

The midgut showed very different host-microbial relationships than the mouthparts. Both *GOX* and *SOD* expression (*CuZnSOD* and *MnSOD*) were strongly and positively associated with abundance of the core five hindgut phylotypes in the midgut. The strongest positive relationship within this core group was *Snodgrassella alvi,* a microaerophilic bacterium important for gut function. In contrast to the mouth, Enterobacteriaceae and ROS gene expression were positively correlated in the midgut. Because both the core hindgut bacteria and Enterobacteriaceae increased in the midguts of aging bees during winter, total bacterial load in the midgut was also positively associated with ROS gene expression. The expression of both *SOD* genes were strongly and positively correlated with the group of five core hindgut bacteria, as was *GOX*, a pro-oxidant widely considered the primary social immune gene (Supplementary Table S4).

Relative to β-*actin*, the expression of two of three measured AMPs increased significantly with age, *Hymenoptaecin* and *Abaecin* (Supplementary Table S4). Most notably, not one of the five core hindgut bacteria was significantly associated with AMP expression on the mouth or in the midgut consistent with their long-term co-evolution with honey bee defensive peptides. However, *defensin-1* was negatively correlated with *L. kunkeei* abundance on the mouth and in the midgut. *Abaecin* expression was negatively correlated with *B. apis* abundance in the midgut. Both *Hymenoptaecin* and *Abaecin* were positively associated with the abundance of Actinomycetales on the mouthparts (Supplementary Table S4).

### Experiment 2: overwintering climate microbiota

**Figure 4:**
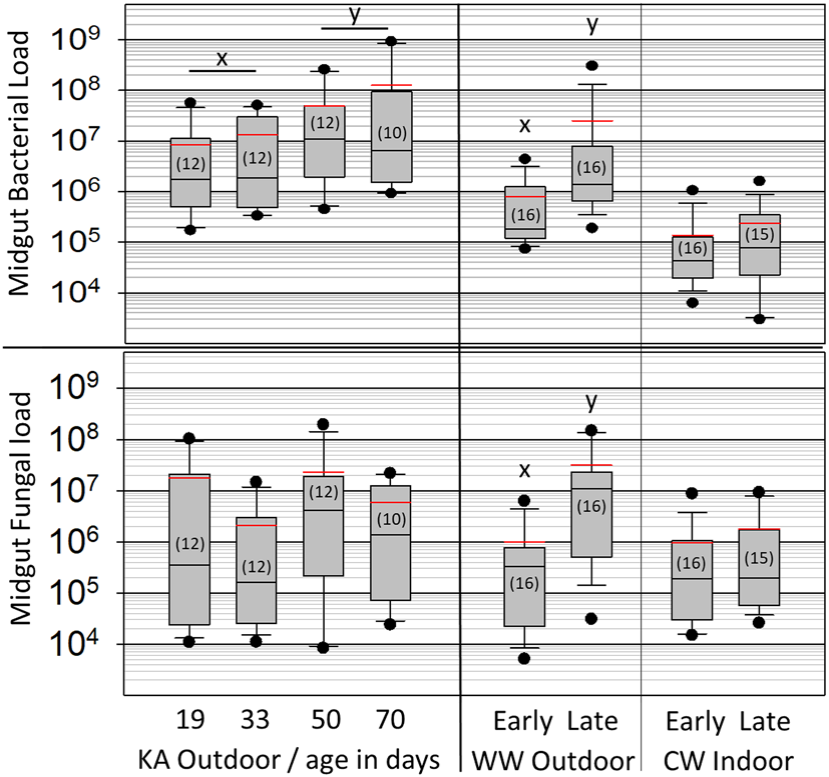
Bacterial and fungal load in the worker midgut of over wintering honey bees. The y-axis is log10 transformed and represents 16S rRNA copy number (bacterial load) and 18S rRNA copy number (fungal load). The WW samples differed significantly from early to late winter for both fungal load (W_32_ = 42, *p* = 0.0008) and bacterial load (W30 = 43, *p* = 0.003). See “Methods” for more sampling details. Grey boxes contain 50% of the variation, whiskers contain 90%, and the dots represent the range. The horizontal red bar is the mean and black is the median. Bacterial load corresponds to microbiotas in Fig. 3 (KA) and Fig. 5 (WW/CW). Sample sizes in parentheses. Boxplots created with SigmaPlot (Systat Software, San Jose, CA).

We sequenced the midgut microbiota of worker bees kept outdoors in southern Arizona or indoors in Idaho at 7 °C and 25% RH. These samples are referred to throughout the manuscript as the WW and CW samples (Fig. 1, Experiment 2), and are the same individuals sequenced for ileum and rectum microbiota in a companion manuscript^8^. Based on the total number of unique 16S rRNA gene sequences, or amplicon sequence variants (ASVs) found in each midgut, bacterial diversity increased significantly from early to late winter in the WW environment (Fig. 2, t_30_ = 2.89, *p* = 0.008, μ = 59 early, 154 late), and decreased significantly in the CW environment; (Fig. 2, t_32_ = 2.89, *p* = 0.007, μ = 253 early, 94 late). Both bacterial and fungal load from early to late winter remained unchanged in CW midguts, but increased significantly in WW midguts (Fig. 4, Bacteria: W_30_ = 43, *p* = 0.003, Fungi: W_30_ = 42, *p* = 0.0008). Fungal load was positively associated with diversity abundance, and both factors increased significantly during winter in WW bees and decreased in CW bees.

**Figure 5:**
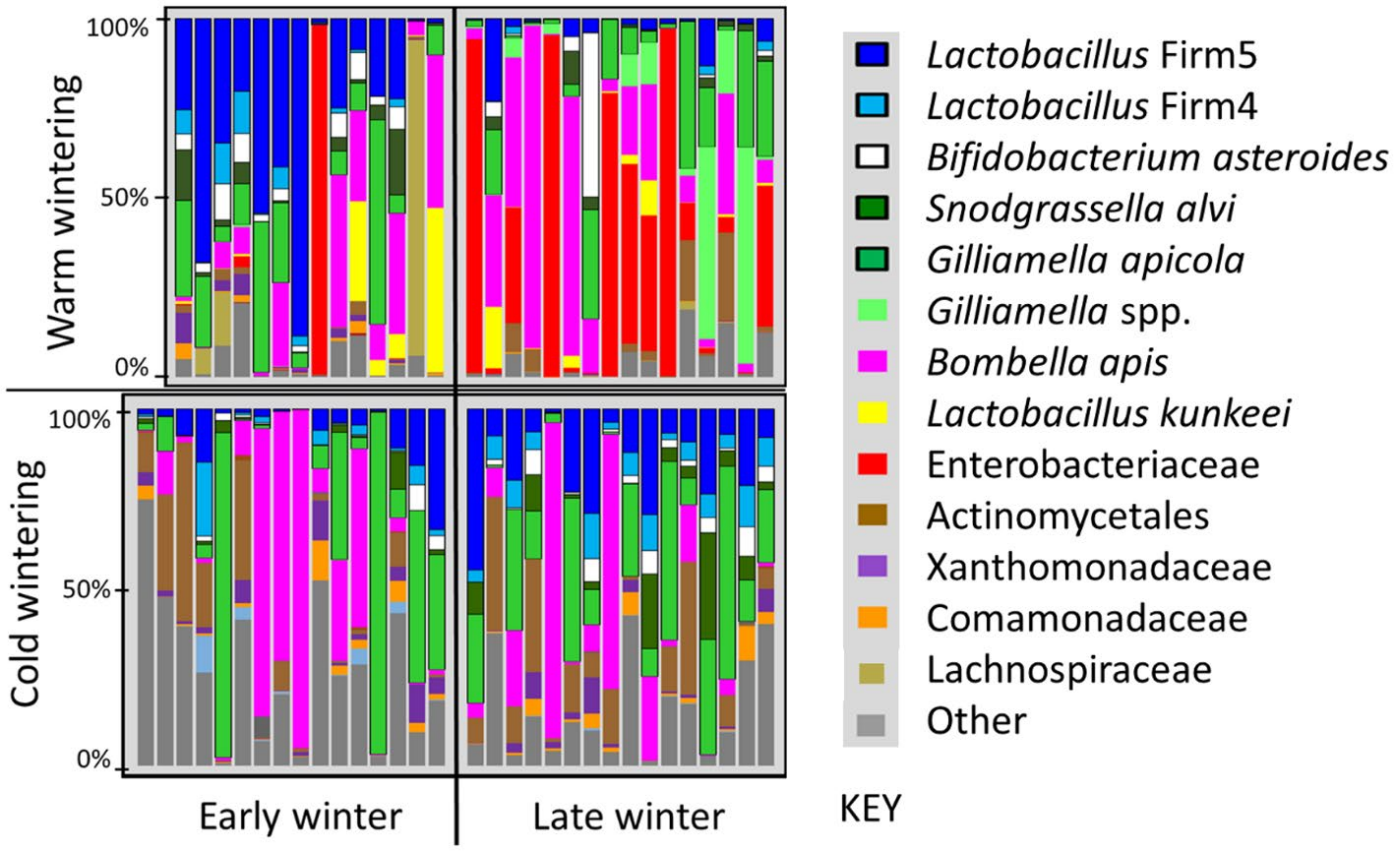
Relative abundance of midgut microbiotas (KEY = OTUs) in early and late winter of worker bees from colonies placed in an indoor climate-controlled facility: “Cold wintering”, or outdoors in Southern Arizona, USA: “Warm wintering”, on the y-axis. Each vertical bar is the midgut microbiota of a single bee from a different colony (*n* = 14 or 16). Outlined in bold within the key, the core five hindgut bacteria declined significantly in the warm outdoor environment, but increased significantly in the cold indoor environment. Significantly larger midgut microbiotas are associated with Enterobacteriaceae (RED) and *Gilliamella* spp. (GFP green). See Supplementary Table S1 and results for more details.

Although separated by 1000 miles, WW and CW midgut environments were comprised of the same OTUs, including all five core hindgut phylotypes, *Bombella apis*, *L. kunkeei,* Actinomycetales, Xanthomonadaceae, and *Delftia* (Comamonadaceae), The latter three OTUs had 100% prevalence and relatively even abundance across both groups (Fig. 5). Core hindgut bacteria increased significantly in the midguts of CW bees over winter, but decreased in WW bees (Fig. 5, Supplementary Table S1).

**Figure 6:**
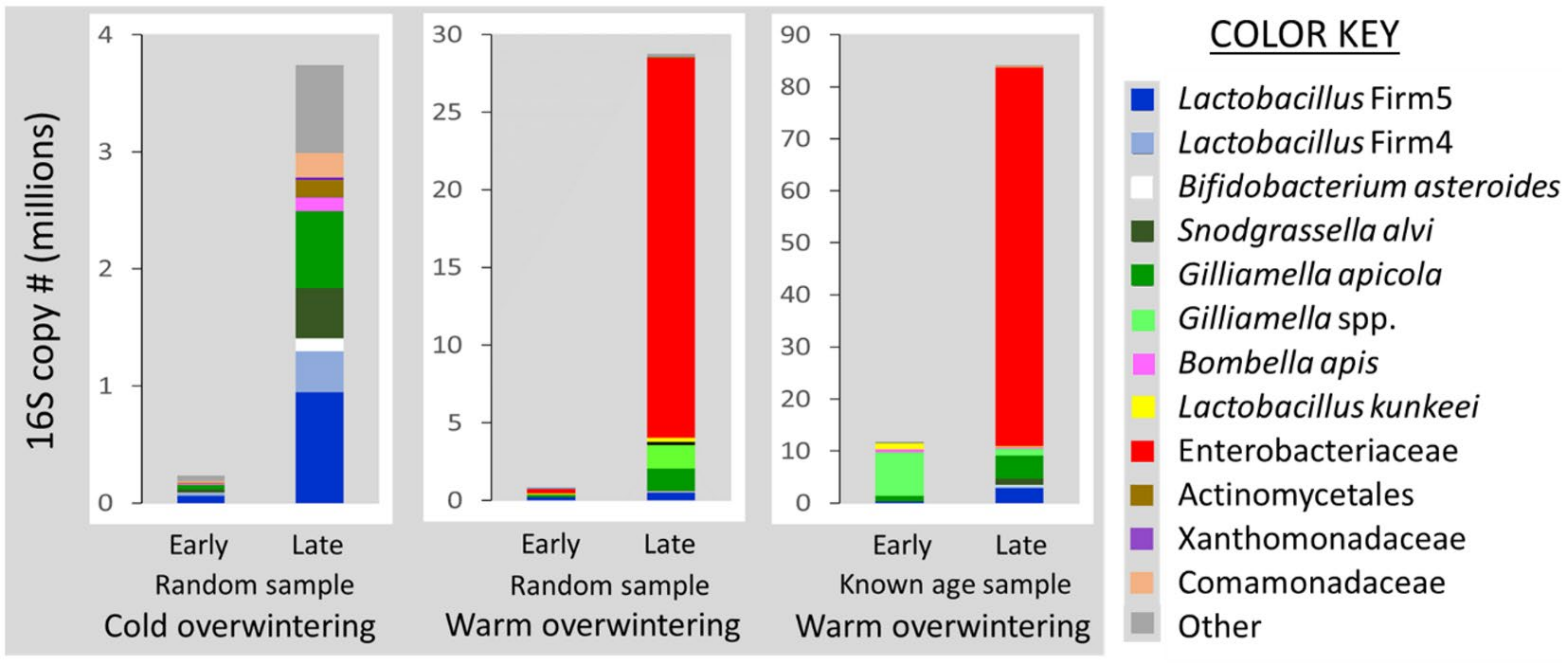
Mean normalized abundance of the midgut microbiota by overwintering environment and sample set. Worker bees were sampled in early or late winter. Random samples are in-hive worker bees of unknown age selected from around the brood nest (*n* = 16 per time point). Known age samples are aged 19/33 days (early, *n* = 16) and 50/70 days (late, *n* = 22). Normalized abundance values for each y-axis are in millions and differ significantly by sample set. See “Methods” for detailed information.

The midgut microbiota from early to late winter differed by overwintering climate (Fig. 5). The CW midguts did not differ from early to late winter when comparing OTU abundance normalized by bactquant and corrected for multiple comparisons (Supplementary Table S1). In CW samples, *L. firm5* and *L. firm4* increased significantly in ratio abundance from early to late winter, and the other core bacteria trended in the same direction (Fig. 5). From early to late winter, the CW colonies experienced a loss of diversity and a loss of non-core bacterial abundance (Figs. 2 and 5). While the CW microbiota changed very little in terms of abundance and composition, colonies experiencing a warm outdoor environment changed drastically for both measures (Figs. 5 and 6).

Rare or absent in the CW samples, Enterobacteriaceae and an unknown *Gilliamella spp.* were abundant in the midguts of late WW samples (Figs. 5 and 6). Increasing in absolute abundance from early to late winter (WW) were Enterobacteriaceae, an unnamed *Gilliamella* spp. (3% different from either *G. apis* or *G. apicola*), *Gilliamella apicola*, *Bombella apis*, Actinomycetales and non-core diversity abundance (Supplementary Table S1). Enterobacteriaceae and the *Gilliamella* spp. accounted for the vast majority of the bacterial increase in midgut microbiota size from early to late winter (Fig. 6). Although at low abundance in WW midguts, Xanthomonadaceae decreased significantly during winter. MANOVA comparisons indicate phylotypes L. Firm5 and L. Firm4 decreased relative to the total microbiota. However, the WW microbiota increased 10X in size during winter (Fig. 6), such that the absolute cell numbers of L. Firm5 did not differ between treatments, but the ratio abundance of L. Firm5 relative to the whole community decreased significantly (Fig. 5). L. Firm5 and L. Firm4 decreased significantly in WW midguts during winter when considered as a proportion of the community whole, as did the less abundant OTUs; Xanthomonadaceae and *Delftia* (Comamonadaceae). Enterobacteriaceae, *Gilliamella *spp. and Actinomycetales increased in normalized abundance, and relative to the total community (Supplementary Table S1).

Associated with small mouthpart microbiomes, Actinomycetales was present in every midgut of the CW samples. Moreover, the CW samples had a signature of three inter-correlated OTUs associated with small, presumably healthy midgut microbiotas; Xanthomonadaceae, Actinomycetales, and *Delftia*, a Commomonadaceae. These bacteria appear in most next generation sequencing datasets, and can also be abundant in the HPG and royal jelly.

Levels of *Nosema ceranae* differed by overwintering climate. Similar to many other microbial metrics, levels of *N. ceranae* remained unchanged from early to late winter in the CW samples. In the WW samples, *N. ceranae* increased significantly from early to late winter. Biologically, *N. ceranae* was at low abundance during winter in both environments based on perceived colony health, a low Ct positive control and high Ct values or non-amplification of many experimental samples (Supplementary Table S2).

### Experiment 2: overwintering climate gene expression

In the CW indoor environment, HPG expression differed from early to late winter for two of seven genes (Table 1). *Glucose oxidase*, and *Defensin-1* expression decreased significantly from early to late winter. The remaining genes either decreased slightly in expression or remained unchanged. Gene expression was associated with host microbial metrics. HPG expression of *Defensin-1* decreased concurrently from early to late winter with bacterial diversity in CW midguts (Supplementary Table S4). *GOX* expression decreased as *S. alvi* midgut abundance increased. For consideration independent of corrected *p*-value in the CW samples, *S. alvi* was positively associated with total bacterial cell abundance, and *GOX* expression was negatively associated with the abundance of fungi, *Bifidobacterium asteroides*, and *G. apicola* in the midgut. *Catalase* expression was negatively associated with bacterial diversity in the midgut (Supplementary Table S4).

In the WW outdoor environment, HPG expression differed from early to late winter for two of seven genes (Table 1, Supplementary Table S3). As a marker of nutritional state, *vitellogenin* expression decreased significantly from early to late winter, while *Abaecin* expression increased. *Vitellogenin* expression was negatively correlated with late winter increases of bacterial diversity, non-core bacterial abundance, and fungal abundance. *Abaecin* expression was negatively associated with Xanthomonadaceae abundance in the midgut from early to late winter, but positively associated with Enterobacteriaceae (Supplementary Table S4). *Hymenoptacin* and *Defensin-1* expression decreased markedly, but not significantly from early to late winter (Supplementary Table S3). Both genes were negatively correlated with the midgut increase in *Nosema ceranae* abundance. *Hymenoptacin* expression was negatively correlated with bacterial diversity, fungal abundance and Actinomycetales. Oxidative state interactions associated with the WW microbiota were limited to *Nosema ceranae* and bacterial diversity, which became more abundant in late winter midguts concurrent with a significant decrease in *GOX* expression from the social gland (Table 1, Supplementary Table S4).

## Discussion

We describe a novel relationship in honey bees involving the mouth, midgut and hindgut microbiota, and social gene expression of aging workers during winter forage dearth. We propose that the hypopharyngeal gland (HPG) performs a vital defensive role during forage dearth in winter, rivaling its established role in providing shared nutrition for colony members during periods of growth. Our results reveal associations of microbiota and social immunity by overwintering environment and highlight the potential role of HPG secretions in mitigating the mouthpart microbiota and shaping the hindgut microbiota during winter. Our results are similar to host-microbial dynamics seen in other social insect species including host secretions that nurture defensive symbionts and reduce colony pathogens^2,6,47,65^. We suggest one mechanistic hypothesis for the lack of immune genes in the honey bee genome^66^; the introduced and resident social microbiota may be mitigated in part by the redox potential of the individual and the group^1,27^.

Our results correspond with those presented in a companion paper that deep sequenced the ileum and rectum microbiotas from the same three (KA, CW, WW) sample sets presented here^8^. It is clear from these and other results that physiologically distinct gut niches are altered somewhat synchronously by microbial opportunism or perturbation^8,14^. Fungal abundance and bacterial diversity decreased significantly and collectively on the mouthparts, ileums and rectums of aging (KA) bees^8^ (Figs. 2 and 4), and showed a strong negative association with the expression of *GOX* and both *SOD* genes from the HPG. Resulting gene expression relative to the general microbial character and taxonomy of all four alimentary tract niches suggests a systemic host-microbial dynamic throughout the entire alimentary tract.

All cold winter (CW) colonies survived the winter, produced the diutinus phenotype, and showed a broad collection of factors that distinguish them from the two warm winter apiaries. The entire CW gut microbiota (midgut, pylorus/ileum, rectum) was either improved from early to late winter or not diminished (Fig. 5)^8^, fungi remained low and constant throughout the entire alimentary tract (Fig. 4)^8^, and bacterial diversity throughout the gut decreased significantly (Fig. 2). The expression of genes associated with oxidative stress and microbial control remained constant in the HPG; both *defensin-1* and *GOX* production decreased significantly over winter consistent with a stable social environment and little microbial challenge (Table 1); similar to other findings from healthy hives kept in cold winter conditions^67^. The abundance of *Nosema ceranae*, a destructive midgut parasite, also remained low and constant in the midgut from early to late winter. The same two bacterial opportunists detected in warm winter apiaries also occurred in CW samples, but at negligible prevalence and abundance, decreasing from early to late winter in the worker hindgut^8^.

In contrast, colonies kept in warm winter conditions (KA and WW) did not produce an overt diutinus phenotype, with only 10% of workers surviving beyond 50 days of age^8^. In practice, both longevity and *Vitellogenin* rich metabolism are indicative of diutinus workers. During winter forage dearth in the southern climate, we documented unique gene expression associated with worker longevity (Table 1, KA samples). The *Vg* levels of randomly sampled workers (WW samples) from late winter were significantly depleted, while *Vg* levels of much older workers from the same climate conditions remained unchanged in late winter, perhaps an indicator of conservation physiology and incomplete expression of the diutinus phenotype. While the average longevity is too low for diutinus bees, we cannot conclude whether the social immune gene expression associated with longevity and higher *Vg* expression is somewhat diutinus in nature (Table 1).

Independent of the diutinus distinction, results from the warm winter sample sets suggest microbial opportunism and disease susceptibility that varied by age cohort. The midguts of both KA and WW treatment groups were overgrown with Enterobacteriaceae and *Gilliamella* spp. in late winter (Fig. 6). The mouthparts of the oldest (KA) bees showed a significant reduction in fungal load with age (Fig. 4), and this pattern was repeated in the hindgut (ileums and rectums) of these same bees^8^. In contrast, fungal load, Nosema disease and bacterial diversity increased significantly during winter in the midguts of randomly sampled WW bees (Figs. 2 and 4), concurrent with significant fungal increases recorded for the ileum and rectum^8^. According to our estimates of worker age, this randomly sampled cohort was less than 30-days-old. Based on these results we suggest that longevity-associated physiology and associated HPG gene expression suppresses fungal growth and bacterial diversity at the individual and social level, and may influence the hindgut microbiota over extended time scales.

Host immune response and various microbial metrics differed significantly between KA and WW samples (Table 1). The significant upregulation of *Vitellogenin* (*Vg*), *GOX*, *SODs* and *AMPs* in the HPG of the oldest KA bees is consistent with a social host response to a microbially challenging overwintering environment (Table 1). *Vitellogenin* is an indicator of nutritional state and a potent antioxidant^68^, while superoxide dismutase (*MnSOD,* and *CuZnSOD*) are able to scavenge excess oxidants associated with metabolic demand or microbial challenge and convert them into less harmful molecules^69^. Of these two, only *CuZnSOD* is secreted by the HPG^70^, while increased expression of *MnSOD* is an indication of increased mitochondrial function (Table 1). Produced in response to social role, *glucose oxidase* (*GOX*) is omnipresent throughout social resource space, and is considered an immediate form of social immune response^38^. *GOX* functions as a pro-oxidant (generates reactive oxygen species; ROS) that thwarts microbial growth by converting omnipresent glucose into hydrogen peroxide and gluconic acid throughout social resource space. In general, the core hindgut bacteria appear resilient to *GOX* expression. The abundance of all five core-hindgut bacteria in the midgut were positively associated with the expression of GOX in the HPG (Supplementary Table S4) highlighting a fundamental host-microbial relationship^27^ that likely facilitates hindgut microbiome establishment/maintenance and limits microbial opportunism.

Social immunity from early to late winter differed by age and winter environment; *GOX* expression increased significantly with age in the (KA) samples, remained unchanged in the WW samples, and decreased significantly in the diutinus CW samples (Table 1). The resulting host-microbial dynamics by sample set suggest a fundamental ecological interpretation associated with the control of ROS at the level of the individual and social group. To review, *S. alvi*, is partnered with the metabolism of *G. apicola* in the ileum of a healthy worker host, contributing to the production of an anoxic hindgut environment^27^. We hypothesize that oxidative control of the gut is associated with the increased abundance and structure of the core hindgut phylotypes in the midgut, as modeled for the ileum microbiota^27^. In the cold indoor environment, *GOX* expression decreased significantly, while the abundance of core hindgut bacteria *S. alvi, G. apicola* and *B. asteroides* increased. In fact, all five of the core hindgut bacteria increased significantly as a correlated group in the CW midgut, while less abundant OTUs, and bacterial diversity decreased (Fig. 5). The core five hindgut bacteria are well equipped to exploit low oxygen gut environments and many strains are capable of producing their own *catalase* and *SOD*^71,72^. Several commensal bacteria, like *Lactobacillus* species, secrete ROS into their surroundings, and this activity discourages the growth of other commensal organisms including fungi. The collective results indicate that CW colonies possessed a healthy microbiota, including low fungal abundance and low microbial diversity, and did not require a social immune response.

In the warm southern environment, *GOX* and *SOD* expression increased significantly from early to late winter in the HPG of the oldest workers (KA samples), associated with a significant reduction in fungal abundance and bacterial diversity on the mouthparts. Older KA worker bees had a strong immune response and low levels of fungal infection and bacterial diversity, while younger bees (WW) sampled at the same time from the same environment appeared more susceptible to dysbiosis. In the WW samples, comprised mostly of middle-aged bees born during the winter, *GOX* production was unchanged from early to late winter, concurrent with significant increases in *Nosema ceranae*, bacterial diversity and fungal abundance. Although both WW and KA midguts were dysbiotic in late winter, the significant decrease in *Vg* expression of WW bees in late winter suggests they could not afford to mount an immune response. Although much older at 50–70 days of age, the long-lived (KA) workers possessed the molecular resources to mount a social immune response to bacterial and fungal opportunism in late winter (Table 1). We hypothesize that many of the microbial differences between older and younger cohorts in late winter involve social and individual immune expression, particularly the control of reactive oxygen species.

Changes in HPG metabolism also suggest that older bees are mitigating microbial growth on the mouthparts. Concurrent with increased upregulation of *GOX, SOD, Abaecin* and *Hymenoptacin* in (KA) bees from early to late winter, the abundance of Xanthomonadaceae (an aerobe), fungal load, and bacterial diversity all decreased significantly on the mouthparts (Fig. 3, Table 1). Although *Hymenoptacin* and *Abecin* are active against a variety of gram negative and enteric pathogens including Enterobacteriaceae^73^, their expression in the HPG was unassociated with the majority of bacteria in the midgut or mouth of (KA) bees. Most notably, none of the core-five hindgut bacteria were significantly associated with AMP expression, consistent with their long-term co-evolution with secreted honey bee defensive peptides. *Abaecin* is highly effective against *Xanthomonas campestris*, a well-known plant pathogen^74^. Known age bees showed a significant reduction of Xanthomonadaceae throughout the gut^8^ from early to late winter. The same Xanthomonadaceae OTU is prevalent and abundant in the microbiota of the HPG and jelly according to two studies^14,18^. This indicates a host response targeted towards a resident colony opportunist but requires further study. Under the same conditions, Actinomycetales became significantly more abundant on the mouthparts. Actinomycetes are typically anaerobic and well known for antibiotic richness, and the suppression of fungal growth^75^. There is a wide variety of native and relatively unknown Actinobacteria in the social environment of honey bee colonies^22^. The associations of this particular Actinobacterial OTU with host-microbial metrics differed by sample set and niche, suggesting it is an influential member of the mouthpart (colony) microbiota with a close association to colony fungi.

The honey bee midgut is a likely target for opportunistic fungi and bacteria in southern climates during winter. Regardless of age, we discovered that the midgut microbiota can be volatile in late winter harboring 10^5^–10^6^ bacterial cells when healthy, but 10^7^–10^9^ cells when dysbiotic (Fig. 6). These midgut microbiotas showed two general enterotypes; smaller microbiotas with somewhat even distributions of 5–9 OTUs and significantly larger microbiotas dominated by one or two OTUs (Fig. 6). Based on absolute abundance, Enterobacteriaceae was the dominant bacterium in the midgut of both outdoor southern experiments sampled in late winter regardless of age or host gene expression. Enterobacteriaceae are part of the native social microbiota, but are not core gut bacteria and many are associated with disease and winter dearth^76^. In both the KA and WW samples, blooms of Enterobacteriaceae and an undescribed *Gilliamella* spp. dominated the midgut microbiota of many individuals and colonies (Fig. 6). The abundance of these two putative opportunists was strongly inter-correlated (Rs = 0.78, *p* = 0.0001), and both were strongly associated with significant increases of total gut fungi and bacterial diversity abundance throughout the entire gut from early to late winter^8^. The hindgut (both ileum and rectum) of these same KA and WW samples also experienced a significant increase of the same two OTUs, but they were < 1% of the hindgut microbiome cell count^8^, suggesting the hindgut was largely uncompromised. The relationship of host gene expression with these two putative opportunists was similar to that for the pervasive midgut pathogen *Nosema*, supporting the hypothesis of opportunism.

The general relationship of oxidative stress with the honey bee gut and social microbiome merits further study. Oxidative stress can either deter or encourage opportunistic infection^77^, and may become difficult to manage in the midgut of aging workers during winter dearth. Our results show that the Enterobacteriaceae OTU detected by this study can evade honey bee defenses on the mouth and crop, then proliferate in the aging worker midgut (Fig. 6). Labeled the honey bee assassin^78^, *Serratia marcescens* was abundant in the midguts of two 70-day-old worker bees. While our midgut dominant Enterobacteriaceae OTU is undescribed, a variety of Enterobacteriaceae are found consistently throughout the social environment. Among these, *Serratia marsescens* and *Enterobacter* spp. are demonstrated chitinolytic^78^, which may provide a growth advantage once they attain the midgut environment. Similar to glucose oxidase GOX in honey bees, the NOX /DUOX enzyme system plays a key role in midgut mucosal immunity of *Drosophila* and *Anopheles* by generating ROS^79,80^. This site-specific response prevents the overproliferation of midgut opportunists, and similar factors may influence microbial balance of the honey bee at both the individual and social level.

## Conclusion

Our results suggests that the midgut of late winter bees is vulnerable to microbial opportunism following an age associated transition in physiology, and if not countered by host gene expression, such opportunism may contribute to dysbiosis and premature senescence of workers and colonies. There are many potential explanations for the host-microbial interactions presented here. We have highlighted one parsimonious perspective evoking social dynamics in response to environmental conditions. Our hypotheses are supported by demonstrated host behavior and physiology^32,81,82^ and a growing understanding of beneficial, opportunistic and pathogenic honey bee microbes in the gut and social environment^7,13,83^. We note that host niches and associated microbiotas are far more complex than discussed, and that detailed experimentation is required to test hypotheses generated by this study.

Our findings provide a novel perspective on health and disease in the social group context. Climate controlled indoor wintering was remarkably stable according to our metrics, suggesting that all sampled worker bees had effectively transitioned to the diutinus phenotype. In contrast, colonies kept outdoors in mild winter environments did not express the diutinus phenotype, and suffered dysbiosis that proliferates primarily in the midgut. Workers born during the winter in southern environments were susceptible to opportunism, while older workers funded by fall nutrition were expressing genes associated with individual and social immunity^82,84,85^. At the colony level, the cost/benefit ratio associated with immune gene expression may be a major factor in surviving mild winters outdoors. Ultimately, our findings suggest that plasticity of lifespan in honey bees was a major factor shaping host-microbial evolution at both the individual and colony level.

## Supplementary Information

Supplementary Information 1.

Supplementary Information 2.

Supplementary Information 3.

Supplementary Information 4.

## Data Availability

Next gene sequencing libraries of the mouth and midgut were deposited in GenBank under Sequence Read Archive (SRA) accession PRJNA742765. Associated with the same KA WW and CW sample sets, next gene sequencing libraries for the ileum and rectum were deposited in GenBank under Sequence Read Archive (SRA) accessions PRJNA705676 (WW and CW samples) and PRJNA705672 (KA samples).
